# Fracture risk and the use of a diuretic (indapamide sr) ± perindopril: a substudy of the Hypertension in the Very Elderly Trial (HYVET)

**DOI:** 10.1186/1745-6215-7-33

**Published:** 2006-12-19

**Authors:** Christopher J Bulpitt, Ruth Peters, Jan A Staessen, Lutgarde Thijs, Marie-Christine De Vernejoul, Astrid E Fletcher, Nigel S Beckett

**Affiliations:** 1 Faculty of Medicine, Imperial College London, UK; 2 University of Leuven, Herestraat 49, Belgium; 3 Hospital Lariboisiere-Paris, Paris, France; 4 London School of Hygiene & Tropical Medicine, London, UK

## Abstract

**Background:**

The Hypertension in the Very Elderly Trial (HYVET) is a placebo controlled double blind trial of treating hypertension with indapamide Slow Release (SR) ± perindopril in subjects over the age of 80 years. The primary endpoints are stroke (fatal and non fatal). In view of the fact that thiazide diuretics and indapamide reduce urinary calcium and may increase bone mineral density, a fracture sub study was designed to investigate whether or not the trial anti-hypertensive treatment will reduce the fracture rate in very elderly hypertensive subjects.

**Methods:**

In the trial considerable care is taken to ascertain any fractures and to identify risk factors for fracture, such as falls, co-morbidity, drug treatment, smoking and drinking habits, levels of activity, biochemical abnormalities, cardiac irregularities, impaired cognitive function and symptoms of orthostatic hypotension.

**Potential results:**

The trial is expected to provide 10,500 patient years of follow-up. Given a fracture rate of 40/1000 patient years and a 20% difference in fracture rate, the power of the sub study is 58% to detect this difference at the 5% level of significance. The corresponding power for a reduction of 25% is 78%.

**Conclusion:**

The trial is well under way, expected to complete in 2009, and on target to detect, if present, the above differences in fracture rate.

## Background

Fragility fractures are associated not only with trauma but with a reduced bone mineral density (BMD), a low level of calcium intake, low concentration of vitamin D in the blood and a high loss of calcium in the urine [1-3]. A high loss of calcium in the urine is also associated with stone formation in the renal tract probably with calcium phosphate as the core ingredient. Hypertensive subjects are particularly vulnerable to calcium stone risk [4,5].

The prevention of osteoporosis and an increase in BMD is achieved therapeutically by increasing calcium intake, increasing vitamin D intake and production in the skin, increasing bone formation or by inhibiting resorption of bone.

Diuretics, such as thiazides increase the passive absorption of calcium in the proximal renal tubules [6] and therefore reduce calcium excretion [7]. It has been suggested that calcium is reabsorbed owing to a reduction in extracellular volume [8]. The effects are employed therapeutically in the prevention of renal stones and benefits have been shown in 3 trials [9-11] with one failing to find a benefit [12]. Indapamide, a sulphonamide related to the thiazide diuretics also reduces calcium excretion and renal stones [13-18]. Two randomised trials suggest that the use of a thiazide diuretic may preserve BMD [19,20] and observational studies suggest they may reduce the incidence of fracture [21-24] but not all studies support this view [25] Results from a meta-analysis suggested that long-term thiazide use may be protective against hip fracture but was not conclusive (OR 0.82, 95% CI 0.62–1.08) [26]. The situation is complicated by the fact that a high oral sodium intake also increases urinary calcium excretion [27]. It has been suggested that indapamide SR may also prevent fractures and indapamide SR is the first line treatment in the placebo controlled Hypertension in the Very Elderly Trial (HYVET) [28]. A sub-study was developed to determine whether or not indapamide prevents fractures in these elderly (≥80 years) hypertensive subjects. The HYVET trial is designed to follow-up subjects for 10,500 patient years. In their meta-analysis, Jones *et al *concluded that a trial of a diuretic to reduce hip fractures by 20% at the 5% level significance in men and women over the age of 70 in the lowest quintile of femoral neck density would require 7,000 patient years with 80% power [26]. The HYVET trial includes subjects only over age 80 and covers all types of fracture but does not include densitometry to identify a low quintile of bone density. This article describes the protocol for the first randomised placebo controlled trial sub study that investigates whether or not indapamide 1.5 mg sustained release reduces the incidence of fractures, a secondary endpoint in the HYVET trial.

### Protocol

#### Brief overview of the HYVET main trial protocol

The HYVET main trial randomises patients with an average systolic blood pressure (over 2 months on placebo) of 160–199 mm Hg and a diastolic pressure <110 mm Hg. Patients must be over the age of 80, have a standing systolic pressure ≥140 mm Hg and with no evidence of renal failure or co-morbidity requiring anti-hypertensive treatment. The full protocol, including all inclusion and exclusion criteria has been published [28]. On randomisation patients are treated using the double-blind method either with indapamide SR 1.5 mg or matching placebo. If goal blood pressure (<150 mm Hg systolic and <80 mm Hg diastolic) is not achieved perindopril 2–4 mg or matching placebo is added as appropriate. The patients are followed-up both according to clinical need and every 3 months in the first year and every 6 months thereafter. At each of these visits data are collected on blood pressure and any events that have occurred since the previous visit. Serious adverse effects are reported as they occur. Blood tests, including serum electrolytes and blood glucose, and quality of life assessments are made annually. The patient may be followed up for a period of 6 months to seven years, depending on date of entry to the trial. The trial has approval of the National and Local Ethical Committees as required.

### Protocol for HYVET Fracture Sub-Study

The trial documents are designed to maximise the reporting of fractures, to identify those factors that are recognised as strongly associated with fracture incidence and to validate the occurrence of any reported fracture.

#### 1. The ascertainment of fractures

The entry form asks for details of all fractures in the previous 20 years including the site of fractures and year of occurrence. This past history may not be complete owing to failure to recall an event. The interim forms (completed after 3,6,9, months and hen six monthly) and the annual forms (completed each year from 12 months until the end of the study) ask for diseases or events since the last visit, including fractures or operations, and is expected to provide almost 100% ascertainment of fractures in those who remain in follow-up.

#### 2. The identification of risk factors for fractures

##### a) Falls

The Interim and annual forms ask 'Has the patient fallen since the last visit?' and 'If yes how many falls?'.

##### b) Associated co-morbidity

The entry form asks for current diseases and typical responses include rheumatoid arthritis and osteoarthritis of the hips. The form also asks for other serious past diseases and past major operations. The interim and annual forms ask for diseases or events since the last visit.

##### c) Associated drug treatment

The entry form asks for the drugs currently being taken including daily dose and year started. The interim and annual forms ask for current drugs taken including aspirin, other analgesics and laxatives etc.

It is expected that drugs associated with falls such as anxiolytics [29] and anti-depressants [30] will be identified by these questions, although these drugs may not be associated with a low BMD [31].

##### d. Symptoms

Symptoms such as light headedness and dizziness may be associated with falls and fractures. Information on these are specifically addressed in the Quality of Life (QOL) forms administered at baseline and annually thereafter. The QOL sub study currently involves 65% of subjects. At baseline and on the interim and annual forms the investigators are also asked to record any symptoms reported by the patients.

##### e. Smoking and drinking habits

Smoking is associated with vascular disease that may precipitate falls and alcohol has a direct effect in inducing falls. Details of smoking habits including number of cigarettes consumed per day, and the number of alcoholic drinks consumed per week are recorded at baseline and on the annual forms.

##### f. Activity

The amount of activity undertaken and the necessity for help during activity are both related to the probability of a fall. The living arrangements, for example in sheltered accommodation, are recorded on the entry, interim and annual forms. Activities of daily living (ADLs) are recorded on the entry and annual forms and include the necessity for assistance with bathing, dressing, going to the toilet and walking along the street.

##### g. Biochemical abnormalities

Biochemical abnormalities such as a low serum sodium are related to falls [32] and are recorded at entry and annually. Blood glucose is also recorded and a high concentration may relate directly to falls or via retinopathy or neuropathy.

##### h. Heart block and atrial fibrillation

Patients with heart block and irregularities of cardiac rhythm have a greater propensity to fall. A standard electrocardiographic tracing is recorded at baseline and annually thereafter to determine these abnormalities and the incidence of myocardial infarction and other cardiac events.

##### i. Impaired cognitive function

Subjects with an impaired cognitive function are recognised to be more at risk of falls [33]. At entry to the trial and annually thereafter the Orientation Memory Concentration (OMC) [34] and Mini Mental State Examination (MMSE) [35] test results are recorded to determine cognitive function. These tests are supplemented by the Clock Drawing Test [36] and Geriatric Depression Scale [37] assessments contained in the QOL forms.

#### 3. Power of the sub-study

Maravic *et al *[38] have reported the incidence of limb fractures leading to hospitalization in French men and women over the age of 81 occurring in 2001. They considered fractures of the hip, proximal humerus and distal radius and/or ulna. The fracture rate for women ≥ 81 years was 2475/million for the humerus, 4256/million for the radius/ulna and 25,846/million for hip fracture. This equates to roughly 3.3%/year. Assuming many fractures of the radius or ulna will not require hospitalization the fracture rate may well be in the region of 4%/year in women. The corresponding figure in men appeared to be about 1.5%/year. In a prospective population based study in Rotterdam the annual incidence of all non -vertebral fracture in subjects aged 80 to 85 years old was 2% in men and more than 3% in women. The fracture incidence was still increased in the subjects that were aged more than 85 years old reaching around 3% in men and 5% in women. [39]. As two thirds of HYVET subjects are women the peripheral fracture rate may be expected to be in the region of 40/1000/year.

The incidence of vertebral fractures in the very elderly is unclear. The annual incidence of vertebral deformity in the Rotterdam study in subjects aged more than 75 was 1.96% in women and 0.9% in men [39]. However in the HYVET trial we do not have radiology of the spine at baseline and it will be difficult to evaluate incident vertebral fractures. We have excluded vertebral fractures from our calculations.

Assuming a fracture rate of 40/1000/years (excluding vertebral fractures) and a 20% difference in fracture rate as suggested by Jones *et al *[26], 10,500 patient years (the target for the main HYVET trial) should detect this reduction at the 5% level of significance and a power of 58%. A 25% reduction would be detected with a power of 78%. These estimates may be conservative as the incidence of fragility (low trauma) fractures may be increasing [40,41] possibly independently of the ageing of the population [40].

#### 4. Validation of Fracture Events

The End Point Committee examines reports of fractures and supplementary information such as radiology reports or operation notes. The Committee (constitution given in the acknowledgements) validates fracture reports as

i) Fracture – validated

ii) Probable fracture unconfirmed – unable to confirm.

iii) No fracture – error in diagnosis

## Discussion

The HYVET sub-study on fractures is the largest placebo-controlled trial of thiazide type diuretic treatment in the prevention of fractures to have been started to date. The risks and benefits of anti-hypertensive treatment in the very elderly (over age 80) hypertensive has yet to be determined. The results of the pilot trial [42], namely that for every stroke prevented there was one extra non-stroke death agree with the results of the INDANA meta-analysis [43]. Thus, if the main trial should show both risks and benefits of treatment, other outcomes may have to be considered. These include any reduction in fracture rate, and any improvement (or deterioration) in cognitive function and quality of life. The very elderly may (or may not) have a greater interest in the prevention of fractures, strokes, and cognitive impairment than in the prolongation of life. The HYVET trial and its sub-studies therefore aim to give a rounded picture of what are the benefits and risks of anti hypertensive treatment in the very elderly, specifically those with mild to moderate hypertension who do not have heart failure or certain other complications of hypertension. The fracture sub-study is an integral part of the complete picture although not the primary end-point of the HYVET trial. The strengths of the sub-study are the inclusion of a high risk (very elderly) group and the attempts to gain full ascertainment and validation of fracture events. A possible, and as yet unconfirmed, weakness is that subjects may be recruited who are of above average fitness for their age group and thus lacking the expected fracture incidence. If this proves true then a trial needs to be performed in patients at even higher risk, for example the very elderly with previous fractures, who do not have a 'low' blood pressure. The HYVET study will also fail to detect many vertebral fractures. The Clinical Research Forms do not ask specifically about back pain and there is no routine radiology. We do, however, base our power calculations only on clinically detected peripheral fractures.

There are reasons to expect a benefit in preventing fractures as diuretics reduce the amount of calcium in the urine. In spontaneously hypertensive rats given large doses (1.5 mg/kg/day) of indapamide SR and a high salt intake, urinary calcium was decreased, sodium excretion increased and sodium-induced bone loss was prevented [44]. The studies in man suggest that thiazide diuretics and indapamide SR reduce calcium excretion in the urine by about 40–50% in patients with hypercalcuria and, in a very small study of patients with essential hypertension, reduce calcium excretion by about 20% [16].

It is relevant and interesting that renal stone production has been reduced by the use of diuretics in some trials but not in others. Four positive trials have been reported that employed hydrochlorothiazide [9] bendroflumethiazide [10] chlorthalidone [11] and indapamide 2.5 mg/day [15]. In the latter study stone formation was reduced by 0.2 stones/per patient/per year and the relative risk for remaining stone free with indapamide was 1.5. The HYVET trial, however, is not powered to detect a reduction in the rare event of passing renal stones.

Are there already data to prove that diuretics prevent fractures? To our knowledge there have not been any randomised controlled trials of the effects of diuretic treatment on the rate of fractures. Nevertheless there have been cross-sectional and longitudinal studies that have been summarised in a meta-analysis [26]. These studies suggested a 20% reduction in hip fracture. On the other hand one case control study reported a 60% increased risk of hip fracture [45]. A meta-analysis of longitudinal studies obviously carries more weight than one case control study.

Recently a pharmacoepidemiologic case-control study of the Danish population [22] reported on 64,699 patients over age 40 with fractures. 194,111 controls used less corticosteroids, thyroxine, anxiolytics, antidepressants and diuretics than the cases. Thiazide diuretics were taken by 22% of controls and 23.7% of cases. However the thiazide users were older, more often women, took more of the other drug groups and had had more previous fractures than non-users. Thus for current users of thiazide diuretics, a crude odds ratio of 1.03 for getting any fracture was reduced to 0.90 (95%CI 0.88–0.93) by full adjustment [22]. When only those subjects who had redeemed ≥2000 defined daily dosages of thiazides over a 5 year period were considered (in order to limit the study to those adherent to regular treatment), this consumption was associated with a 26% reduction in any fracture, a 19% reduction in hip fracture, a 5% reduction in fracture of the spine and a 31% reduction in forearm fractures. Moreover a second study has suggested that forearm fractures may be reduced by 37% in those using thiazide diuretics for more than 8 years [24]. The results of a further large pharmacoepidemiological study suggested a 20% reduction in all fractures with the use of thiazide diuretic but also a 23% reduction with the use of beta blockers on their own [21]. This has been confirmed recently but a benefit has also been suggested for angiotensin converting enzyme (ACE) inhibitors and calcium-Channel blockers [46]. This raises the possibility that blood pressure reduction or confounding factors may be responsible for the benefits.

## Conclusion

It is hoped that the HYVET fracture sub-study will provide a clear answer to the benefit or otherwise of diuretic (indapamide slow release 1.5 mg ± perindopril 2–4 mg) treatment in the prevention of fractures in the very elderly hypertensive. It is also possible that the findings of the study will help determine the risks and benefits of treatment and assist in the overall decision: to treat or not treat the very elderly hypertensive subjects with a diuretic ± ACE inhibitor?

## Competing interests

The authors are all involved in the running of the HYVET study. CJB is the lead investigator, AEF the co-investigator, NSB the trial co-ordinator, RP the deputy trial co-ordinator, LT and JAS serve on the Data Monitoring Committee and M.C de V on the End Point Committee. The trial is financed by grants from the British Heart Foundation and the Institut de Recherches Internationales Servier. Thus all authors have received salaries or expenses from Servier, a company that may benefit from the outcome of the HYVET study.

## Authors' contributions

The functions of the authors in the HYVET study are listed above. CJB and AEF designed the main study and the sub-study, with considerable input from the other authors. CJB wrote the first draft; all authors read and commented on the draft and final manuscripts; and all approved the final manuscript.
